# Ginsenoside Rg1 Delays Chronological Aging in a Yeast Model via CDC19- and SDH2-Mediated Cellular Metabolism

**DOI:** 10.3390/antiox12020296

**Published:** 2023-01-28

**Authors:** Siming Wang, Juhui Qiao, Chunyan Jiang, Daian Pan, Shiting Yu, Jingjing Chen, Shichao Liu, Peiguang Zhang, Daqing Zhao, Meichen Liu

**Affiliations:** 1 Northeast Asia Research Institute of Traditional Chinese Medicine, Changchun University of Chinese Medicine, Changchun 130117, China; 2 School of Pharmacy, Changchun University of Chinese Medicine, Changchun 130117, China; 3 Changchun Institute of Optics, Fine Mechanics and Physics, Chinese Academy of Sciences, Changchun 130033, China

**Keywords:** ginsenoside Rg1, *Saccharomyces cerevisiae*, aging, oxidative stress, metabolic homeostasis

## Abstract

Ginsenosides, active substances in *Panax ginseng* C. A. Meyer (ginseng), extend lifespan in multiple species, ameliorate age-associated damage, and limit functional decline in multiple tissues. However, their active components and their molecular mechanisms are largely unknown. Here, ginsenoside Rg1 (Rg1) promoted longevity in *Saccharomyces cerevisiae*. Treatment with Rg1 decreased aging-mediated surface wrinkling, enhanced stress resistance, decreased reactive oxygen species’ production and apoptosis, improved antioxidant enzyme activity, and decreased the aging rate. Proteomic analysis indicated that Rg1 delays *S. cerevisiae* senescence by regulating metabolic homeostasis. Protein–protein interaction networks based on differential protein expression indicated that CDC19, a homologue of pyruvate kinase, and SDH2, the succinate dehydrogenase iron–sulfur protein subunit, might be the effector proteins involved in the regulation by Rg1. Further experiments confirmed that Rg1 improved specific parameters of mitochondrial bioenergetics and core enzymes in the glycolytic pathway. Mutant strains were constructed that demonstrated the relationships between metabolic homeostasis and the predicted target proteins of Rg1. Rg1 could be used in new treatments for slowing the aging process. Our results also provide a useful dataset for further investigations of the mechanisms of ginseng in aging.

## 1. Introduction

Aging is a complex process linked to the gradual loss of physiological integrity and is the major contributing factor to most common chronic human diseases [1]. The population is growing progressively older worldwide, and these extended lifespans present serious problems for healthcare systems. Consequently, there is an urgent need for approaches that promote healthy aging. Much research has focused on natural compounds with anti-aging properties but low toxicity and few side-effects. 

Ginseng (*Panax ginseng* C. A. Meyer) is a traditional medicinal material in East Asia that is highly valued for its wide range of biological activities and pharmacological effects. Ginsenosides—one of the primary active substances in ginseng—exert multiple biological functions and health benefits. Ginsenosides can delay several age-related human diseases, such as cognitive decline, spontaneous tumors, and cardiovascular and immune dysfunction [2], and extend the healthy lifespan of both *Drosophila melanogaster* [3] and *Caenorhabditis elegans* [4]. Ginsenosides are a collection of many saponin monomers, and each monomeric saponin has unique functional activity. For example, ginsenoside Rg1 (hereafter, Rg1) enhances synaptic growth and restores neurotransmitter dysfunction in chronic neuroinflammation rats [5]. Ginsenoside Rb1 can improve learning and memory by increasing the production of acetylcholine [6]. Ginsenoside Rh2 inhibits cervical cancer by regulating cellular energy metabolism [7]. Ginsenoside Rd promotes cardiac repair in myocardial infarction mice by activating the Akt/mTOR signaling pathway and regulating monocyte/macrophage subset conversion [8]. Thus, ginsenosides are good candidate drugs in longevity research. However, which monomer saponins (A component in ginsenosides or a subset of ginsenosides) have the best effects in prolonging life/preventing aging has not yet been determined, and the corresponding mechanisms and targets of the ginsenosides remain unclear. 

The mechanism of aging is complex. High-throughput “omics” technologies have been widely used in aging research. The application of such technologies has unveiled a proteomic aging clock and identified key processes that change with age in different species, including *Drosophila* [9], mice [10], and humans [11]. With the maturity of high-throughput technologies, many new anti-aging targets have been discovered beyond the classical regulatory pathways associated with longevity [12,13]. 

The unicellular eukaryote *Saccharomyces cerevisiae* has cellular characteristics analogous to those of mammals [14]. Some of the most complex life activities can be found in this yeast. The genes controlling meiosis, cell cycle regulation, and DNA repair have high homology with the equivalent human genes [15]. Therefore, *S. cerevisiae* is a convenient and appropriate model organism for studying aging.

The main objective of the present study was to identify saponin monomers that extend the longevity of naturally aging *S. cerevisiae* and gain insight into the associated molecular mechanisms. We designed a comparative proteomic analysis using the Data-Independent Acquisition (DIA) technique combined with bioinformatic analysis to find changes in the *S. cerevisiae* proteome after treatment with a monomeric saponin of interest. We used chronologically senescent *S. cerevisiae* cells to verify the effects of the saponin via changes to colony growth and morphology. The resistance of *S. cerevisiae* cells to stress was also tested, especially antioxidant reactions. Our data suggest that Rg1 has anti-aging effects and regulates homeostasis of energy metabolism by controlling the *S. cerevisiae* homologues of pyruvate kinase (CDC19) and the succinate dehydrogenase iron–sulfur protein subunit (SDH2). We provide a comprehensive dataset for further investigations of the mechanisms of ginseng in aging.

## 2. Materials and Methods

### 2.1. Drugs

The ginsenosides Rb1, Rb2, Rd, Rg1, Rg2, Rg3, Rh1, and Rh2 (cat #: B21050, B21051, B21054, B21057, B21058, B21059, B21061, and B21062) were purchased from Shanghai Yuanye Biotechnology Co., Ltd. (Shanghai, China). 

### 2.2. Yeast Strains and Growth Conditions

The wild-type yeast strain *S. cerevisiae* BY4742 (ATCC^®^ 201389™) was obtained from Miaoling Biotechnology Co., Ltd. (Wuhan, China). Yeast strains were grown in liquid yeast extract–peptone–dextrose (YPD) medium, including 1% (*w*/*v*) yeast extract (Thermo, Belmont, MA, USA), 2% (*w*/*v*) peptone, and 2% (*w*/*v*) glucose, or on solid YPD medium containing 2% agar. Experiments were carried out at 28 °C, unless otherwise stated.

### 2.3. Growth

Yeast cells were incubated in a shaking incubator (180 rpm; Eppendorf AG, Hamburg, Germany) at 28 °C. Growth was monitored by using an Infinite 200 PRO microplate reader (Tecan Trading AG, Männedorf, Switzerland) at 600 nm. 

### 2.4. Colony-Forming Unit Assay

Yeast strains were treated with or without Rg1 (180 μg/mL) and observed after 16, 33, 50, 68, 84, and 90 h of incubation. Cell suspension (5 μL, 1:10,000 dilution) was spread on YPD-agar plates. After incubation for 2 days at 30 °C, colonies were counted [16]. And the OD value was measured using a microplate reader (Tecan Trading AG, Männedorf, Switzerland) at 600 nm.

### 2.5. Scanning Electron Microscopy (SEM)

Yeast cells with or without Rg1 treatment (180 μg/mL) were transferred to a pretreated slide (soaked in 0.1% polylysine at 37 °C for 30 min). After attachment, the yeast cells were fixed with 2.5% glutaraldehyde for 4 h at 4 °C. Then, the slide was washed twice with phosphate-buffered saline (PBS) and kept in a desiccator for 12 h. Finally, it was gold plated to a thickness of 5–10 nm and observed by SEM (FESEM LEO Supra 50 VP, Carl Zeiss, Oberkochen, Germany).

### 2.6. Apoptosis Assay

Yeast cells with or without Rg1 treatment (180 μg/mL) were detected after 16, 33, 50, 68, 84, and 90 h. Apoptotic and necrotic cells were, respectively, labeled with Annexin V-fluorescein isothiocyanate and propidium iodide (Becton Dickinson and Company, NY, USA), then analyzed using a flow cytometer (Amnis Corporation, Seattle, WA, USA) and quantified with IDEAS software v6.1 (Amnis Corporation, Seattle, WA, USA).

### 2.7. Stress Tests

Yeast cells normally cultured for 16, 33, 50, 68, 84, and 90 h were collected. Cells were stressed in the following conditions: NaCl (final concentration 1 M, incubation for 1 h with shaking); H_2_O_2_ (final concentration 5 mM, incubation for 1 h with shaking); acetic acid (0.3% *v*/*v*, incubation for 2–4 h with shaking); and heat shock (incubation at 47 °C for 20–40 min). The stress-treated cells were transferred to the same volume of normal liquid YPD and incubated at 28 °C for 48 h, and then their growth was detected at 600 nm.

### 2.8. Measurement of Antioxidant Biomarkers 

Cells were grown for 16, 33, 50, 68, 84, and 90 h, collected at 2700× *g* for 5 min, and used for measurement. Reactive oxygen species (ROS; cat # S0033S), peroxidase (POD) activity (cat # BC0095), superoxide dismutase (SOD) activity (cat # BC0175), catalase (CAT) activity (cat # BC0205), glutathione (GSH) concentration (cat # BC1175), thioredoxin (TRX) activity (cat # BC1155), and malondialdehyde (MDA) concentration (cat # BC0025) were determined using kits from Solarbio Science & Technology Co., Ltd., Beijing, China, except the ROS kit, which was from Beyotime Biotechnology, Shanghai, China. The details are as follows:

ROS level was determined in yeast cells using 2,7-dichlorofluorescein-diacetate (H_2_DCF-DA). The cells were washed in PBS and inoculated into black 96-well plates, and 10-µL H_2_DCF-DA (20 μM) was added before detection. The cells were incubated at 37 °C for 20 min in dark conditions, and then detected using a fluorescence microplate reader with excitation at 488 nm and emission at 530 nm. 

Total SOD activity: Cells were lysed in phosphate buffer (pH 7.2) ultrasonic crushing (200 W, ultrasonic 3 s, interval 10 s, repeat 30 times). Centrifuged 8000× *g* at 4 °C for 10 min, the supernatant was taken. SOD activity was measured by mixing the cell lysate with 0.1 mM EDTA, 75 μM nitroblue tetrazolium (NBT), 2 μM riboflavin, and 13 mM methionine and the absorbance was measured at 560 nm. Protein content was measured by the Bradford method. The SOD activity is expressed in active units (U)/mg of total protein.

GSH content: Cell lysate was prepared in phosphate buffer and centrifuged (8000× *g* at 4 °C for 10 min). The cell extract was suspended in ice-cold phosphate buffer (pH 7.0) and mixed with an equal volume of ice-cold 2 M HClO_4_ containing 4 mM EDTA. After incubation for 15 min, the mixture was centrifuged and the supernatant was neutralized with 3 M KOH in 2 mL of 100 mM phosphate buffer, and then 50 μL DTNB (5,5′-dithiobis-2-nitrobenoic acid, 10 mM) was added on ice. After incubation for 5 min, the absorbance was measured at 412 nm. 

MDA content: Lipid peroxidation in *S. cerevisiae* was quantified by determining the thiobarbituric acid (TBA)-reactive substance MDA. Cell lysate was prepared in phosphate buffer and centrifuged (8000× *g* at 4 °C for 10 min). To the supernatant, TBA reagent (0.25 M HCl, 15% trichloroacetic acid, and 0.375% TBA) was added and the mixture was heated for 15 min in a boiling water bath. After cooling, the absorbance was measured at 535 nm using a spectrophotometer. The concentration of MDA in samples was calculated using 1,1,3,3 tetramethoxypropane as a standard, and the results are expressed in μM of MDA/mg of total protein.

CAT activity: Cell lysate was prepared in phosphate buffer and centrifuged (8000× *g* at 4 °C for 10 min). The cell lysate (50 μL) was mixed with 30 mM H_2_O_2_ solution (250 μL, in 50 mM PBS, pH 7.0). The change of absorbance at 240 nm was measured continuously over 10 min after adding the H_2_O_2_. One U of CAT activity was defined as the amount of enzyme that gave a decrease in absorbance at 240 nm of 0.01 in 1 min. The results are expressed in U/mg of total protein.

POD activity: Cell lysate was prepared in phosphate buffer and centrifuged (8000× *g* at 4 °C for 10 min). The cell lysate (100 μL), 140 μL 0.3% guaiacol (in 50 mM PBS, pH 6.4), and 60 μL 0.3% H_2_O_2_ (in 50 mM PBS, pH 6.4) were mixed. The absorbance of the reaction solution at 470 nm was measured continuously. The results are expressed in U/mg of total protein.

TrxR activity: TrxR catalyzed the reduction of DTNB by NADPH to generate TNB and NADP+. TNB has a characteristic absorption peak at 412 nm. The increase in rate of formation of TNB at 412 nm can be determined to calculate the activity of TrxR.

### 2.9. Reverse Transcription–Quantitative PCR (RT–qPCR)

Total RNA was isolated from cells using TRIzol reagent [17]. cDNA was synthesized using a kit from Takara Bio Inc. following the manufacturer’s instructions, and reverse transcribed according to the instructions of the Prime Script RT reagent kit (Takara Biotechnology Co., Ltd., Beijing, China). Aliquots of cDNA were subjected to qPCR analysis with SYBR Green PCR Master Mix (Takara Biotechnology Co., Ltd., Beijing, China) and a real-time PCR system equipped with a CFX 96 Connect™ Optics Module (Bio-Rad Laboratories, Inc., Hurcules, CA, USA). The reaction program was predenaturation for 3 min at 95 °C, then 40 cycles of denaturation at 95 °C for 5 s, annealing at 54 °C for 15 s, and extension at 72 °C for 30 s. *Tubulin* was used as the internal reference gene. The PCR primer sequences used are shown in Table 1. Data were analyzed using the 2^−∆∆cq^ method [18].

### 2.10. Establishment of Proteomics Database

Proteomic analysis was performed using two samples (Control and Rg1). All proteins with fold-change (FC) ≥ 1.2 and *p* < 0.05 were considered to be differentially expressed. The expression difference factor between groups and the *p*-value (*t*-test) of up- and downregulated proteins were used as criteria to draw volcano plots. Fisher’s exact test was used to compare Kyoto Encyclopedia of Genes and Genomes (KEGG) pathways for KEGG pathway enrichment analysis in the target protein set. A bubble diagram of KEGG enrichment was drawn using the Rich factor, *p*-value, and the number of genes enriched in the pathway. Direct and indirect interactions between proteins (protein–protein interactions, PPI) were found based on information in the STRING database and IntAct, and CytoScape software was used to generate interaction networks.

### 2.11. Mitochondrial Oxidative Phosphorylation (OXPHOS) and Glycolysis Assays

Oxygen consumption rates (OCR) and extracellular acidification rates (ECAR) were measured using an XFp Extracellular Flux Analyzer (Agilent Technologies, Inc.) according to the manufacturer’s instructions. The probe plate of the Seahorse XFp sensor was preincubated overnight in calibration solution. A total of 4.8 × 10^4^ cells/well (yeast cells pretreated with Rg1) were seeded into XF 8 cell culture plates (Agilent Technologies, Inc., Santa Clara, CA, USA). Cells were washed with 180 µL of Dulbecco’s modified Eagle’s medium and then incubated at 37 °C for 1 h before measurement.

OCR was determined following injection of oligomycin (an ATP synthase inhibitor, 1.5 µM), carbonyl cyanide 4-(trifluoromethoxy) phenylhydrazone (FCCP, a proton-carrying uncoupler, 2 µM), or rotenone (Complex I inhibitors, 0.5 µM) (all using the XFp Cell Mito Stress Test kit (Agilent Technologies, Inc., Santa Clara, CA, USA)). ECAR was determined following injection of glucose (the substrate for hexokinase, 10 mM), oligomycin (5 µM), or 2-deoxy-D-glucose (a competitive inhibitor of hexokinase, 50 mM). The use of these inhibitors permits the determination of key aspects of mitochondrial function [19].

### 2.12. Construction of CDC19-Overexpression and SDH2-Deletion Strains

The strains used in this study are shown in Supplementary Figure S1. The yeast open reading frame (ORF) library was designed for use in high-throughput chemical and genetic screens in *S. cerevisiae*. PCR amplification of ORFs from the library results in addition of directional *attB* sequences directly abutting the initiating ATG codon and the final sense codon of the gene of interest. After two rounds of recombination, an ORF flanked by directional *attB* sequences was Gateway-cloned cloned in-frame into vector pBG1805, with a triple affinity tag comprised of His_6_–HAepitope–3Cprotease site–ZZproteinA. A *cdc19*-overexpressing (*OE*) strain was constructed by transforming yeast cells with *Bsr*GI-digested plasmid BG1805-*cdc19*-*OE*.

A PCR-generated [20,21] deletion strategy was used to systematically replace the open reading frame of SDH2 from its start to its stop codons with a KanMX module and two unique 20mer molecular bar codes. Each deletion cassette was constructed using two sequential PCR reactions. In the first amplification, 74-bp UPTAG (CCCTGCTTAATCATTATGGATGTCCACGAGGTCTCTATATGACCGCCCATGCGTAGCGTACGCTGCAGGTCGAC) and 74-bp DNTAG (CCCGTCGCTATCTCGTCACGGTGTCGGTCTCGTAGCACTAGCTCAATGAGGTTAATCGATGAATTCGAGCTCG) primers amplified the KanMX gene from pFA6-kanMX4 DNA; expression of KanMX means that cells can be selected using gentamicin [22]. In the second PCR reaction, two ORF-specific 45-mer oligonucleotides (UP: TCCAAATACACCTGCCCAGTCTCTAGACCCTGCTTAATCATTATG and DOWN: GCATCAGATAGAAGACTATTTAAGAACCCCGTCGCTATCTCGTCA) were used to extend the ORF-specific homology to 45 bp, increasing the targeting specificity during mitotic recombination of the gene disruption cassette. Then, the PCR products were transformed into *S. cerevisiae* BY4742 to replace SDH2 by homologous recombination, and the transformants were selected on SD URA medium (PerkinElmer, USA). The *sdh2Δ* cells were verified by PCR using primers 5′-ACATCGTAGGAAGTCTGAGC-3′ (forward) and 5′-CATTAGTGCCAAGTCGAGTA -3′ (reverse).

### 2.13. Determination of Core Enzymes in Glycolysis

Hexokinase, 6-phosphofructokinase (PFK), phosphoglycerate kinase (PGK), and pyruvate kinase (CDC19) enzyme-linked immunosorbent assay (ELISA) kits were purchased from Shanghai Preferred Biotechnology Co., Ltd. Yeast cells were pretreated with Rg1, homogenates were centrifuged at 1620× *g* for 10 min, and the supernatant was taken to determine the amounts of hexokinase, PFK, PGK and CDC19. According to the instructions of the ELISA kits, 50 μL of standard was added to the standard well, and the sample wells contained 10 μL of sample and 40 μL of buffer. HRP-labeled antibody (100 μL) was added to the standard and sample wells, and the mixtures were incubated at 37 °C for 60 min. Washing was repeated five times. Then, 100 μL of substrate was added to each well, and the samples were incubated at 37 °C for 15 min in the dark. Stop solution (50 μL) was added, and the samples were detected at 450 nm using a microplate reader (Tecan Group). 

### 2.14. ATP Content

The ATP content of yeast cells was determined using a kit (cat # S0026, Beyotime Institute of Biotechnology, Shanghai, China). Yeast cells were centrifuged at 1060× *g* for 10 min, ATP detection solution (200 µL) was added, and the mixture was centrifuged at 4246× *g* for 5 min at 4 °C. The supernatant obtained after centrifugation was mixed with 100 µL of ATP detection solution. The ATP content was determined using a microplate reader (Tecan Group).

### 2.15. Mitochondrial Membrane Potential (MMP) Analysis

Yeast cells were mixed with Rhodamine 123 (2 µM; cat # R8030, Beijing Solarbio Science & Technology Co., Ltd., Beijing, China) and incubated in darkness at 37 °C for 30 min. After centrifugation at 530× *g* for 5 min, a flow cytometer (Amnis Corporation, Seattle, WA, USA) was used for detection, and IDEAS software v6.1 was used for quantitative analysis.

### 2.16. Statistical Analysis

All data represent at least three independent experiments, evaluated statistically using one-way analysis of variance and subsequent *post-hoc* Bonferroni’s test for behavior. 

## 3. Results

### 3.1. Rg1 Delays Chronological Aging of S. cerevisiae Cells Better Than Other Ginsenoside Monomers 

**Figure 1:**
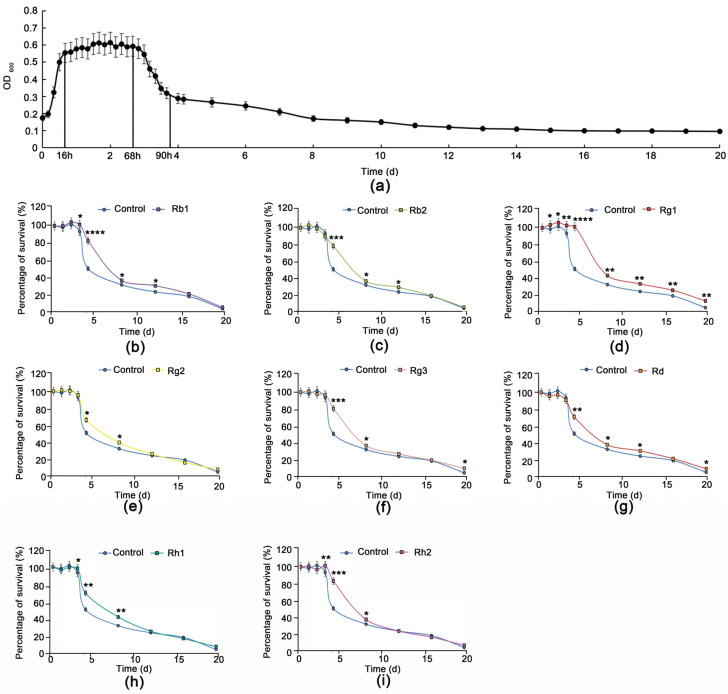
Ginsenosides promote survival of *Saccharomyces cerevisiae* cells. Growth curves of yeast cells without treatment (**a**). Survival percentage curve on treatment with ginsenoside monomers Rb1 (**b**), Rb2 (**c**), Rg1 (**d**), Rg2 (**e**), Rg3 (**f**), Rd (**g**), Rh1 (**h**), and Rh2 (**i**) (all at 180 µg/mL). Data are expressed as the mean ± SD; *n* = 3. Compared with untreated controls, * *p* < 0.05, ** *p* < 0.01, *** *p* < 0.001, **** *p* < 0.0001.

The chronological lifespan (CLS) of yeast cells is the survival time of a certain number of nondividing cells in the stable period. This is a mature model system for human post-mitotic cell senescence [23]. From our growth curve of *S. cerevisiae*, the cells began to enter a stable period at 16 h, which lasted until 68 h. After 68 h, the cells entered the senescent phase (Figure 1a). Thus, the stable period of the *S. cerevisiae* cells was 16–68 h, and intervention with ginsenosides was begun at 16 h. For comparison of the effect of ginsenosides, the CLS of yeast cells was used as the evaluation index.

**Figure 2:**
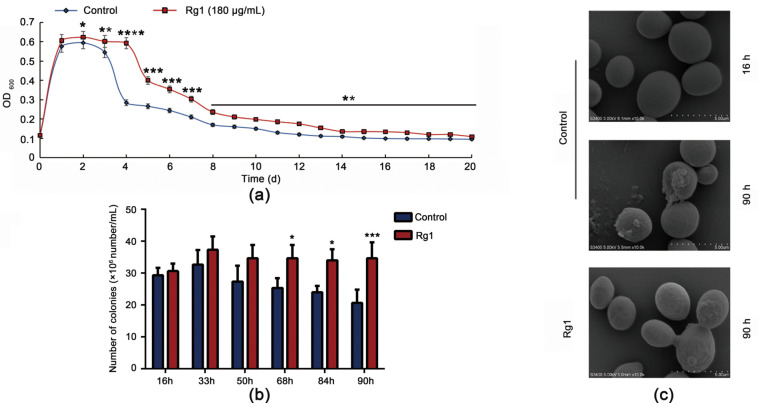
Ginsenoside Rg1 promotes survival and improves the morphology of *S. cerevisiae* cells. (**a**) Growth curves of yeast cells without or with Rg1 (180 µg/mL) treatment. (**b**) Spot number analysis of yeast cells without or with Rg1 (180 µg/mL) treatment. (**c**) Morphological changes of yeast cells under scanning electron microscopy without or with Rg1 (180 µg/mL) treatment. Data are expressed as the mean ± SD; *n* = 3. Compared with untreated controls, * *p* < 0.05, ** *p* < 0.01, *** *p* < 0.001, **** *p* < 0.0001.

To explore which ginsenoside monomer in ginsenosides had the best effect in delaying the senescence of *S. cerevisiae* cells, we tested eight such ginsenoside monomers (Rb1, Rb2, Rg1, Rg2, Rg3, Rd, Rh1, and Rh2). Rg1 had a significant life-extending effect on the yeast cells (Figure 1b–i, Table S1). It prolonged their life when applied at 100–250 µg/mL (Figure S2). The maximal effect was observed at 180 µg/mL Rg1 (Figure 2a), at which the stable period of the yeast cells was prolonged by approximately 24 h, to 96 h. Subsequently, on the basis of qualitative spot phenotypic analysis, we determined that Rg1 promotes yeast cell viability (Figure 2b and Figure S6). Under SEM, at 16 h, the cell morphology was regular ovoid, and the surface was smooth. For untreated cells, at 90 h, cell surface damage, wrinkles and adhesion appeared, resulting in cell rupture, and decreased cell survival rate. Rg1 intervention maintained better cell morphology, reduced cell wrinkling and adhesion, and reduced cell rupture (Figure 2c). To observe how Rg1 results in healthy aging in yeast cells, we used six time points between 16 and 90 h for the subsequent experiments (the cell survival rate was 50% at 90 h).

### 3.2. Rg1 Delays Apoptosis of S. cerevisiae Cells

**Figure 3:**
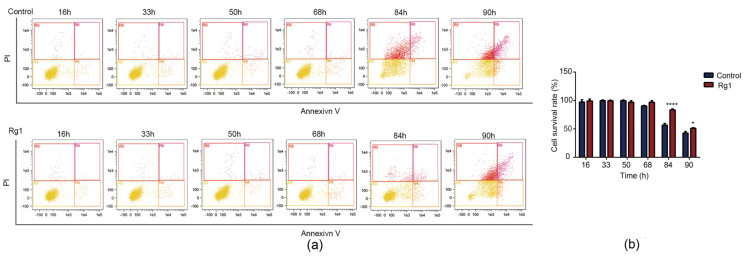
Rg1 delays apoptosis of *S. cerevisiae* cells. (**a**) The percentage of apoptotic cells was detected by flow cytometry. (**b**) Quantification of normal analysis from panel a. Data are expressed as the mean ± SD; *n* = 3. Compared with untreated controls, * *p* < 0.05, **** *p* < 0.0001.

Senescence (68–90 h) increased the proportion of yeast cells in early apoptosis. Rg1 treatment delayed the occurrence of aging-mediated apoptosis (Figure 3a,b). 

### 3.3. Rg1 Increased Stress-Resistance of S. Cerevisiae Cells

**Figure 4:**
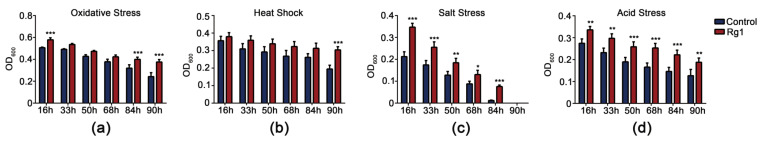
Effect of Rg1 on stress-resistance of *S. cerevisiae* cells. Analysis of yeast cells OD value under oxidative (**a**), high-temperature (**b**), high-salt (**c**), and acid stress (**d**) with or without Rg1 (180 µg/mL) treatment. Data are expressed as the mean ± SD; *n* = 3. Compared with untreated controls, * *p* < 0.05, ** *p* < 0.01, *** *p* < 0.001.

Decreased resistance to external stress is an important feature of aging. Yeast cell stress analysis showed that Rg1 treatment enhanced the resistance of yeast cells to oxidative stress, high-temperature stress, high-salt stress, and acid stress (Figure 4a–d and Figure S7). 

### 3.4. Rg1 Enhances the Antioxidant Defense System to Scavenge ROS in S. cerevisiae

**Figure 5:**
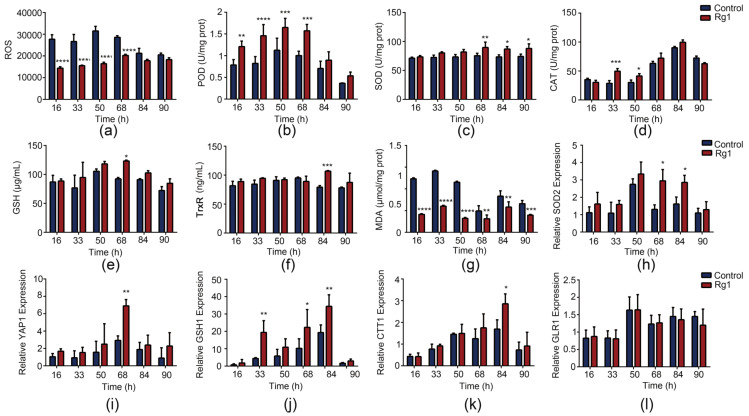
Measurement of intracellular (**a**) reactive oxygen species (ROS) levels, (**b**) peroxidase (POD) activity, (**c**) superoxide dismutase (SOD) activity, (**d**) catalase (CAT) activity, (**e**) glutathione (GSH) concentration, (**f**) thioredoxin (TrxR) activity, and (**g**) malondialdehyde (MDA) concentration with or without Rg1 (180 µg/mL) treatment. Analysis of antioxidant-related gene expression in yeast cells ((**h**), *SOD2*; (**i**), *YAP1*; (**j**), *GSH1*; (**k**), *CTT1*; (**l**), *GLR1*) by qRT–PCR in cells with or without Rg1 treatment. Data are expressed as the mean ± SD; *n* = 3. Compared with untreated controls, * *p* < 0.05, ** *p* < 0.01, *** *p* < 0.001, **** *p* < 0.0001.

The aging free radical theory states that the main means of anti-aging is the elimination of free radicals from the body [24]. Our results showed that Rg1 treatment effectively decreased the level of ROS in yeast cells (Figure 5a). We examined antioxidant enzyme activities and related gene expression levels to confirm the antioxidant benefits of Rg1. The enzyme activities of POD (Figure 5b), SOD (Figure 5c), CAT (Figure 5d), TrxR (Figure 5f), and GSH content (Figure 5e) were elevated after Rg1 treatment. Rg1 also decreased the MDA (Figure 5g) content of the cells. Similarly, the mRNA levels of antioxidant-enzyme-related encoding genes, including *SOD2*, *YAP1*, *GSH1*, *CTT1* and *GLR1*, were notably increased by Rg1 treatment (Figure 5h–l). 

### 3.5. Rg1 Treatment Altered Proteomic Profiles in Aging S. cerevisiae Cells

**Figure 6:**
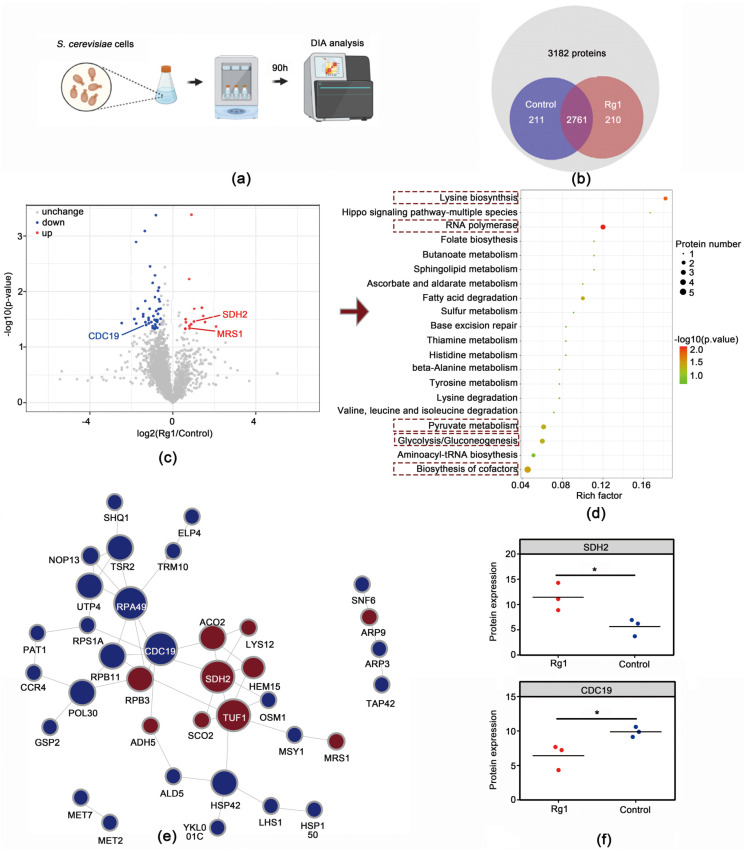
Rg1 treatment altered the proteomic profile of *S. cerevisiae* cells. (**a**) Schematic diagram of the Data-Independent Acquisition proteomic analysis preparation process. (**b**) Venn diagram of proteins enriched in yeast cells. (**c**) Volcano plot of protein expression changes. Control vs. Rg1, “Control” was untreated, and “Rg1” was treated from 16 to 90 h. Differences were considered statistically significant at *p* < 0.05. (**d**) The top 20 enriched Kyoto Encyclopedia of Genes and Genomes (KEGG) pathways. The significance of the KEGG pathways is indicated by the intensity of the red color. (**e**) Protein–protein interaction network analysis of the effect of Rg1 of *S. cerevisiae*. The node color indicates an increase (red) to decrease (blue) of the protein interaction counts. The size of the circle represents the number of proteins interacting with other proteins in the network. (**f**) Changes in expression levels of CDC19 and SDH2 differential proteins (the data were scaled down based on proteomics output data, CDC19 shows data reduced 1,000,000-fold, SDH2 shows data reduced 10,000-fold). Protein levels were quantified and analyzed by one-way analysis of variance. Data are expressed as the mean ± SD; *n* = 3. Compared with untreated controls, * *p* < 0.05.

To determine how Rg1 delays aging, we used DIA proteomic analysis to compare protein expression in yeast cells at 90 h, with and without Rg1 treatment (Figure 6a). In the proteomic analysis, a total of 2761 proteins were identified in the cortices of the yeast cells from the two groups (Figure 6b). Among them, 60 proteins (15 upregulated and 45 downregulated) showed significantly changed expression between the Control and Rg1 groups (proteins with *p* < 0.05 and FC ≥ 1.2 were considered differentially expressed) (Figure 6c, Table S2). Functional characteristics of the differentially expressed proteins were determined using KEGG pathway enrichment analyses. The top 20 enriched pathways are shown in Figure 6d. Among the pathways stimulated by Rg1 treatment were those of RNA polymerase, lysine biosynthesis, biosynthesis of cofactors, glycolysis/gluconeogenesis, pyruvate metabolism, fatty acid degradation, and a variety of metabolic pathways. 

Interaction with other proteins is one of the key ways in which proteins perform their functions. Thus, we performed differentially expressed protein interaction network analysis. The PPI network results showed that CDC19 and SDH2 were highly connected, that is, they interacted directly with multiple proteins in the network (expressed as circle size in Figure 6e). CDC19 is pyruvate kinase, which is involved in glycolysis. SDH2 is a subunit of succinate dehydrogenase, an enzyme that is part of both the tricarboxylic acid (TCA) cycle and the mitochondrial respiratory chain. Protein CDC19 expression was downregulated and SDH2 upregulated by Rg1 treatment (Figure 6f). We thus speculated that Rg1 delays *S. cerevisiae* cell senescence by affecting aging-mediated metabolic disorders, and CDC19 and SDH2 are its main targets. 

### 3.6. Rg1 Downregulates Glycolysis and Upregulates OXPHOS to Promote Longevity of S. cerevisiae Cells

**Figure 7:**
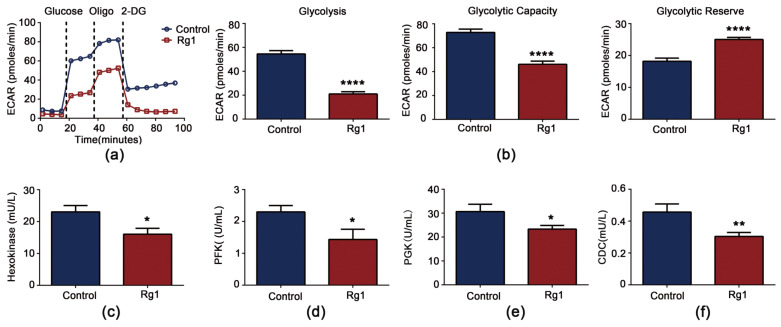
Effects of Rg1 treatment on glycolysis in *S. cerevisiae*. (**a**) Graphical representation of extracellular acidification rate (ECAR) measurements over time. (**b**) Calculation of representative functional parameters of glycolysis from ECAR curves. “Glycolysis” indicates the ECAR when glucose was injected; “Glycolytic Capacity” indicates the ECAR when oligomycin was injected; “Glycolytic Reserve” indicates the difference between Glycolysis and Glycolytic Capacity. Activities of the enzymes hexokinase (**c**), phosphofructokinase (PFK) (**d**), phosphoglycerate kinase (PGK) (**e**), and CDC19 (**f**) detected by using enzyme-linked immunosorbent assay kits. Data are expressed as the mean ± SD; *n* = 3. Compared with untreated controls, * *p* < 0.05, ** *p* < 0.01, **** *p* < 0.0001.

Metabolism is intimately connected with aging and lifespan regulation [25], and DIA proteomic analysis suggests that it is associated with Rg1 prolonging the lifespan of *S. cerevisiae* cells. An XFp extracellular flux analyzer was used to assess the effects of Rg1 on glycolysis and OXPHOS, the two major energy metabolism pathways. Both the basal and stimulated ECAR of *S. cerevisiae* decreased after Rg1 treatment (Figure 7a). Rg1 significantly decreased the ECAR linked to glycolytic capacity and the glycolytic reserve in yeast cells (Figure 7b). 

As with pyruvate kinase (CDC19) (Figure 7f), we observed that the core glycolytic enzyme activities of hexokinase (Figure 7c), PFK (Figure 7d), and PGK (Figure 7e) were decreased by Rg1 treatment. RT–PCR analysis confirmed that Rg1 inhibited expression of key glycolytic enzyme-encoding genes (Figure S3). We drew similar conclusions from KEGG enrichment pathway analysis, in which Rg1 reversed the expression levels of triose-phosphate isomerase (an enzyme between hexokinase and PFK in glycolysis) and PGK in aged *S. cerevisiae* cells (Figure S4). Overall, Rg1 inhibits glycolysis in *S. cerevisiae*.

**Figure 8:**
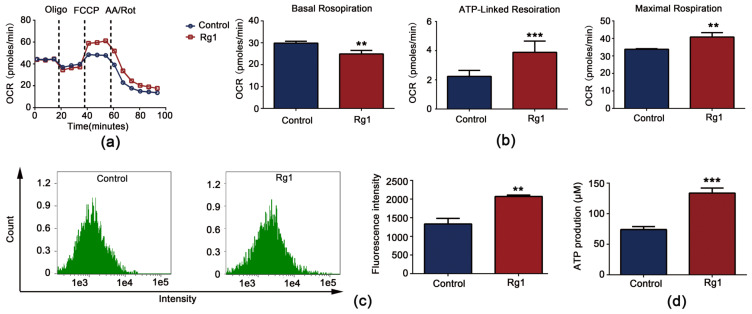
Effects of Rg1 treatment on mitochondrial respiration. (**a**) Graphical representation of oxygen consumption rate (OCR) measurements over time. (**b**) Calculation of representative functional parameters of OXPHOS from OCR curves. “Basal Respiration” indicates the OCR in the absence of oligomycin; “ATP-linked Respiration” indicates the OCR after injection of oligomycin; “Maximal Respiration” indicates the OCR after injection of FCCP. (**c**) Mitochondrial membrane potential (MMP). (**d**) ATP production. Compared with untreated controls, ** *p* < 0.01, *** *p* < 0.001.

Treatment of cells with Rg1 (180 µg/mL) recovered the senescence-induced decrease in the OCR, suggesting that the OXPHOS capacity was increased (Figure 8a). Yeast cells had a significantly increased OCR linked to ATP production and maximum respiratory capacity following Rg1 treatment (Figure 8b). Succinate dehydrogenase plays a central role in OXPHOS (it is Complex II of the electron transport chain). The mRNA expression of the succinate dehydrogenase complex component SDH2 was significantly upregulated by Rg1 (Figure S3). In yeast cell chronological aging studies, the MMP was found to gradually decrease with aging, indicating that the loss of MMP leads to cellular energy depletion or cell senescence [26]. Rg1 recovered the senescence-induced decrease in MMP (Figure 8c). MMP maintains the physiological function of the cellular respiratory chain and ATP production. ATP levels were increased after Rg1 treatment of yeast cells (Figure 8d). Together, these results indicate that Rg1 supplementation balances energy metabolism. 

### 3.7. CDC19 and SDH2 Are Required for Rg1 to Regulate Glycolysis and OXPHOS to Delay Aging

**Figure 9:**
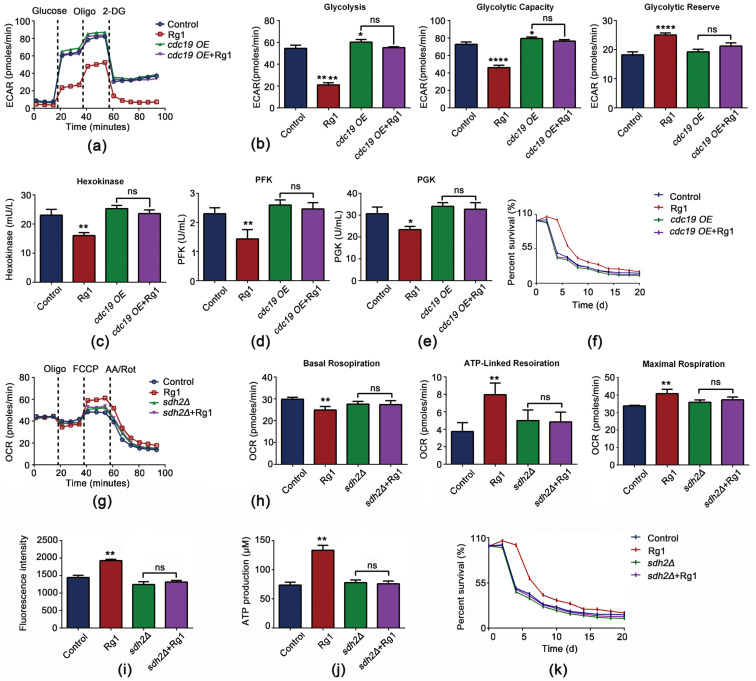
Antisenescence effects of Rg1 intervention on *CDC19*-overexpressing (*cdc19 OE*) and *SDH2*-deficient (*sdh2Δ*) strains of *S. cerevisiae*. (**a**) Graphical representation of ECAR measurements over time in *S. cerevisiae* strain BY4742 (the parental strain; Control and Rg1) and the *cdc19 OE* strain. (**b**) Calculation of representative functional parameters of glycolysis from ECAR curves. “Glycolysis” indicates the ECAR when glucose was injected; “Glycolytic Capacity” indicates the ECAR when oligomycin was injected; “Glycolytic Reserve” indicates the difference between Glycolysis and Glycolytic Capacity. Hexokinase (**c**), PFK (**d**), and PGK (**e**) enzyme activities. (**f**) Growth curves of *S. cerevisiae* BY4742 (Control and Rg1) and *cdc19 OE* strains. (**g**) Graphical representation of OCR measurements over time in strains BY4742 (Control and Rg1) and *sdh2Δ*. (**h**) Calculation of representative functional parameters of OXPHOS from OCR curves. “Basal Respiration” indicates the OCR in the absence of oligomycin; “ATP-linked Respiration” indicates the OCR after injection of oligomycin; “Maximal Respiration” indicates the OCR after injection of FCCP. MMP (**i**) and ATP (**j**) production levels in *S. cerevisiae* strains BY4742 (Control and Rg1) and *sdh2Δ*. (**k**) Growth curves of strains BY4742 (control and Rg1) and *sdh2Δ*. Data are expressed as the mean ± SD; *n* = 3. Compared with untreated controls, * *p* < 0.05, ** *p* < 0.01, **** *p* < 0.0001.

To confirm that SDH2 and CDC19 are the main targets by which Rg1 regulates glycolysis and OXPHOS and so delays aging, we established *CDC19*-overexpressing (*cdc19 OE*) and *SDH2*-deficient (*sdh2Δ*) *S. cerevisiae* strains. First, we observed that after *CDC19* overexpression, glycolysis was increased (Figure 9a,b), and the activities of hexokinase (Figure 9c), PFK (Figure 9d), and PGK (Figure 9e) were also increased (no statistical significance). Although Rg1 treatment of the *cdc19 OE* strain slightly reduced the activities of hexokinase, PFK, and PGK, it did not affect glycolysis overall, and Rg1 treatment did not increase the longevity of the *cdc19 OE* strain (Figure 9f). 

Next, we tested whether the Rg1-mediated temporal delay of senescence in yeast cells was affected by *SDH2* deficiency. Consistent with previous studies, ATP (Figure 9i) and MMP (Figure 9j) levels were significantly decreased in both aging and *SDH2*-deficient cells, indicating that SDH2 may be associated with aging. Rg1 intervention failed to increase ATP production or the decrease MMP level in *SDH2*-deficient cells, and thus OXPHOS was not increased (Figure 9g,h), and Rg1 treatment did not increase the longevity of *sdh2Δ* cells (Figure 9k). We also found that overexpression of *CDC19* or deletion of *SDH2* counteracted the protective effect of Rg1 against oxidative stress (Figure S5). On the basis of these results, we suggest that the life-extending effect of Rg1 on yeast cells is based on regulation via CDC19 and SDH2.

## 4. Discussion

Analyzing the interaction between aging characteristics and anti-aging drugs is a major challenge in current research on aging. The ultimate goal is to find effective anti-aging drugs that can improve human health during aging and have long-term efficacy with few side-effects. Studies ranging from single-celled yeast to animal model systems have shown that aging mechanisms are conserved and controlled by a highly interconnected and functionally complex network of gene and protein interactions [27]. *S. cerevisiae* has been used widely as a research model in aging studies to help understand the molecular mechanisms of the anti-aging properties of natural compounds [28,29]. CLS refers to the survival time of a certain number of nondividing yeast cells in the stable phase, and it is the most intuitive indicator for evaluating aging and screening potential anti-aging drugs [30]. 

We observed that the lifetime of *S. cerevisiae*, as grown in this work, was divided into a growth phase (0–16 h) and a stable phase (including a plateau phase (16–68 h) and a decay phase (68 h onward)). During the period 0–16 h, the individual morphology and physiological indexes of the yeast were not stable, and it was not suitable for cell sampling and retention [31]. Therefore, 16 h was chosen as the starting point for the intervention in this study. The traditional Chinese herbal medicine ginseng has been proven to have good anti-aging effects [32]. Our previous research and other studies have shown that ginsenoside mixtures can significantly prolong the healthy lifespan of *Drosophila* and *C. elegans*. In the present study, we selected monomeric saponins that are widely used in anti-aging [33] and for age-related diseases [34]. We compared eight ginsenoside monomers and found that Rg1 significantly prolonged the lifespan of yeast cells, with the maximum elongation observed on treatment with 180 μg/mL Rg1. Interestingly, the plateau phase of yeast cells was significantly prolonged, by approximately 24 h, on intervention with 180 µg/mL Rg1, which therefore extended the plateau to 96 h. The extension of CLS largely depends on the extension of the plateau phase and was similar to the effect of certain drugs on life extension [35,36]. Furthermore, qualitative spot and SEM analyses confirmed that Rg1 increased the survival ratio of yeast cells and avoided the surface wrinkling and fracture caused by aging of the cells. To observe how Rg1 results in healthy aging in yeast cells, we used six time points between 16 and 90 h for the subsequent experiments (the cell survival rate was 50% at 90 h).

Senescence is usually characterized by apoptosis, growth cycle arrest, decreased proliferation, and abnormal expression of genes [37]. Here, senescence increased the abundance of yeast cells in early apoptosis, while Rg1 treatment significantly delayed the occurrence of apoptosis [38]. The ability to withstand external pressure is an important indicator of body health [39]. The results of yeast cell spot analysis showed that Rg1 treatment enhanced the resistance of cells to oxidative, high-temperature, high-salt, and acid stress. Aging is related to the gradual accumulation of oxidative stress, which damages cell function and reduces survival [40]. The effects of ROS-mediated oxidative stress are balanced by antioxidant systems, including enzymatic and nonenzymatic antioxidants. The most important antioxidants and free radical scavengers responsible for ROS elimination include SOD, CAT, POD, and GSH. Our results showed that Rg1 lowered ROS levels in yeast cells, and increased the activities of antioxidant enzymes (POD, SOD, CAT, and TrxR), the expression levels of related genes (including *SOD2*, *YAP1*, *GSH1*, *CTT1*), and the concentration of GSH. Elp3 has been reported to participate in the oxidative stress response of *S. cerevisiae* by changing the nuclear accumulation and activity of transcription factor *YAP1*, which regulates the expression of antioxidant genes [41]. Rg1 affected two redox systems related to oxidative stress in *S. cerevisiae*, TrxR and GSH, helping to control the redox state of cells by preventing and repairing damage to molecules prone to oxidation. We hypothesize that Rg1 may alleviate oxidative stress through the transcription factor YAP1, mediating related antioxidants and ROS scavengers. Consistent with previous studies [42], ROS induced apoptosis in aging yeast cells, indicating that antioxidant defense plays an important role in induced apoptosis. The *SOD1*, *SOD2*, and *FIS1* genes of yeast cells are homologous to those in humans, and mutation or change of expression of these genes in humans is related to neurodegenerative diseases [43]. In addition, studies in yeast have indicated that human diseases associated with defects in antioxidant and antiapoptotic genes can be treated by pharmacological interventions [44]. Here, Rg1 enhanced the activity of antioxidant enzymes, decreased apoptosis, and decreased the rate of aging.

Restoring the normal activity of disrupted gene expression and signaling pathways through pharmacological intervention is the most promising approach to improving healthy aging [45]. Proteomic analysis showed Rg1-mediated changes in protein expression in *S. cerevisiae* were closely related to glycolysis/gluconeogenesis, pyruvate metabolism, fatty acid degradation, and a variety of metabolic pathways. The differentially expressed protein interaction network showed that Rg1 treatment significantly downregulated CDC19 and upregulated SDH2. CDC19 is a key rate-limiting enzyme in glycolysis, while SDH2 (a succinate dehydrogenase subunit) is part of the TCA cycle and the mitochondrial respiratory chain. Aging is characterized by a dysregulated metabolism with upregulation of glycolysis and downregulation of OXPHOS [46]. In consequence, we speculate that Rg1 delays *S. cerevisiae* cell senescence via the regulation of senescence-mediated glycolytic and OXPHOS metabolic disorders.

Specifically, the enzyme activities of hexokinase, PFK1, and PGK increase during aging, indicating that overactive glycolysis is a metabolic feature of senescent cells [47]. Studies have reported that in primary human brain microvascular endothelial cells [48] and aging diabetes [49], transcriptional changes in metabolic enzyme genes increased the activity of the glycolytic pathway and, in the latter case, influenced the function and characteristics of pancreatic β-cells. Disruptions to mitochondrial OXPHOS are associated with aging and neurodegeneration [50]. For example, mitochondria in older animals cannot produce a sufficient energy supply because of a decrease in OXPHOS. In *C. elegans* [51] and *D. melanogaster* [52], OXPHOS intermediate mutation accelerates aging and shortens lifespan. Thus, metabolic interventions can be viewed as promising strategies to promote longevity and to prevent or delay age-related disorders. In this study, the effect of Rg1 on aging required the normal expression of intermediates (such as CDC19 and SDH2) involved in the glycolysis and OXPHOS pathways, that is, maintaining metabolic balance. Compared with aged yeast cells, Rg1 treatment lowered glycolytic capacity, the glycolysis rate, and key enzyme activity, consistent with *Curcumae Radix* [28] and sulforaphane [53], which inhibited senescence-associated overactive glycolysis. During OXPHOS, the OCR measured in the presence of inhibitors targeting the mitochondrial electron transport chain was increased by treatment with Rg1. SDH2 is part of the mitochondrial respiratory chain and is required for OXPHOS. The observations that Rg1 interacts with SDH2, and the increased expression of many mitochondrial-related genes in cells treated with Rg1, demonstrates a direct functional role of Rg1 in mitochondrial respiration. Future work should focus on exploring the bias of ATP production between glycolysis and OXPHOS in the aging process.

To demonstrate the effect of CDC19 and SDH2 in the regulation of CLS in *S. cerevisiae*, we constructed *cdc19 OE* and SDH2-deficient strains. In the *cdc19 OE* strain, the activity of core enzymes in the glycolytic pathway was increased compared with that in parental strain BY4742. We hypothesize that in the *cdc19 OE* strain, the consumption of enolpyruvate might be increased, the core enzyme activity in the glycolytic pathway increased [47], and the glycolytic pathway promoted, which accelerates aging. On disruption of SDH2, the MMP decreased, and the mitochondria were unable to produce a sufficient energy supply, which is the result of weakened oxidative phosphorylation, as occurs in mitochondria of aged animals [54]. Our results confirm that CDC19 and SDH2 are closely related to aging. We also observed that the mutations in the *cdc19 OE* and SDH2-deficient strains affected the effect of Rg1 to a certain extent. That is, Rg1 did not exert longevity benefits in these mutant strains. These findings further suggest that the glycolytic and OXPHOS metabolic status maintained via CDC19 and SDH2 are pathways via which Rg1 exerts anti-aging effects.

## 5. Conclusions

**Figure 10:**
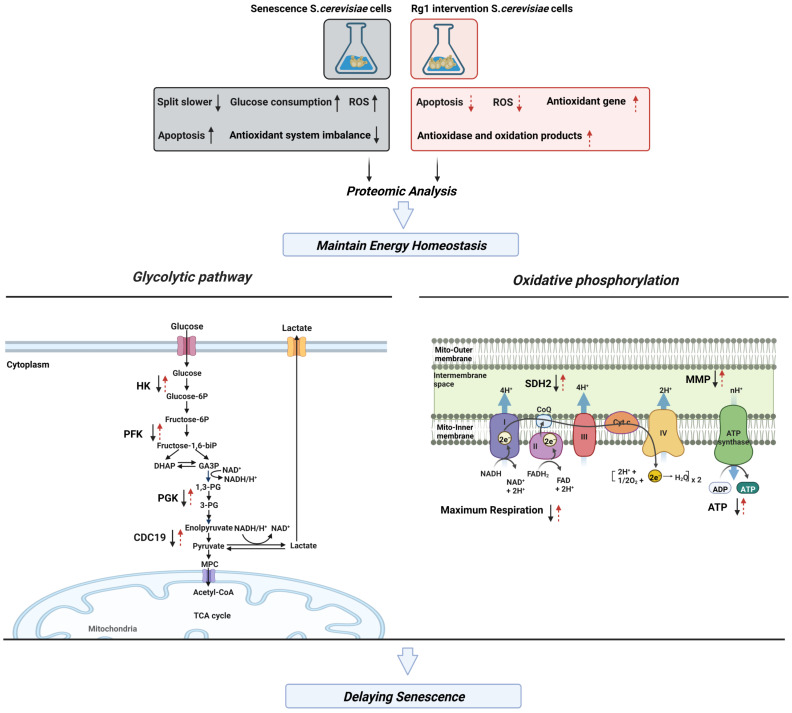
The effects of Rg1 on prolonging the chronological lifespan of yeast cells may be summarized as follows: improvement of specific parameters of mitochondrial bioenergetics, maintenance of energy metabolism homeostasis, enhancement of the activity of antioxidant enzymes, inhibition of the production of ROS, and decreased apoptosis. Rg1, ginsenoside Rg1; ROS, reactive oxygen species; HK, hexokinase; PFK, phosphofructokinase; PGK, phosphoglycerate kinase; CDC19, homologue of pyruvate kinase; ATP, adenosine triphosphate; SDH2, succinate dehydrogenase iron–sulfur protein subunit; MMP, mitochondrial membrane potential.

In summary, Rg1 can prolong the CLS of yeast cells. It restores cellular morphology and improves the cell survival rate. It also enhances the activity of antioxidant enzymes, inhibits the production of free radicals, and decreases apoptosis. On the basis of DIA analysis and mutant strains, Rg1 maintains energy metabolic homeostasis; its main targets are CDC19 and SDH2. These results suggest that regulating energy metabolism may be a potential form of intervention to resist aging and age-related diseases (Figure 10). Our data provide new insights into the mechanism by which ginsenoside Rg1 delays aging.

## Figures and Tables

**Table 1:** Primer sequences for RT–qPCR.

Gene	GenBank Accession	Primer Sequence (5′–3′)
Tubulin	NM-001179929	F: CCAAGGGCTATTTACGTGGA
		R: GGTGTAATGGCCTCTTGCAT
SOD2	NM-001179138	F: GCATTACACCAAGCACCATCA
		R: CCAGGTTTTCCCAGAATAGACA
CTT1	NM-001181217	F: TCATCACCCATACGCTTCT
		R: GGACATTTGTAACCCACATTCT
GSH1	NM-001181534	F: GCTGTTCGTGCTTACAAGTGAC
		R: ATGCCTCCAAATCCGTTCT
YAP1	NM-001182362	F: GACGGCGTGGGGAAGAAGGC
		R: CCGACATCCAGGCGGCGTTT
GLR1	NM-001183905	F: TATTTGGATGGGCTAGATTC
		R: TTCAACATTACCGTCCTTATT
PFK	NM-001181369	F: GATGCTTCTGGGTTCCGTAT
		R: ACCTTGACTTTGAGCCTTGT
CYC1	NM-001181706	F: GGTTCTGCTAAGAAAGGTGCTA
		R: CCTTCAGCTTGACCAGAGTG
CDC19	NM-001178183	F: AGAAGAACCTCCATCATT
		R: AGACTTGTGGTATTCGTA
PGK	NM-001178725	F: TGTCTTGGCTTCTCACTTGG
		R: TTCGTTTCTTTCACCGTTTG
NDI1	NM-001182483	F: ATCATTATCTGCCGTTAGCCA
		R: CAAATGTGTTAGGTTCCGCA
FIS1	NM-001179415	F: AGTCCCGTAGACGAGAATGC
		R: CCACCTGCTTGTTATTACGCT
TPI	NM-001180358	F: AACTTTCTTTGTCGGTGGTA
		R: TTCCTTAATGGATTGTTTGG
HXK	NM-001181119	F: AAAACCACAAGCCAGAAAGG
		R: GGGAAATCCATAACCCAACC

## Data Availability

Data is contained within the article and Supplementary Material.

## Supplementary Materials

The following supporting information can be downloaded at: https://www.mdpi.com/article/10.3390/antiox12020296/s1, Figure S1: Yeast strains used in this study; Figure S2: CLS curve of yeast cells with Rg1 treatment at 100–250 μg/mL; Figure S3: RT–qPCR analysis of expression of glycolysis genes in yeast cells; Figure S4: Expression levels of triose-phosphate isomerase and PGK in KEGG enrichment pathway analysis; Figure S5: Overexpression of *CDC19* and deletion of *SDH2* counteracted the protective effect of Rg1 against oxidative stress; Figure S6: Spot representation of yeast cells without or with Rg1 (180 µg/mL) treatment; Figure S7: Speckle phenotype analysis under oxidative (a), high-temperature (b), high-salt (c), and acid stress (d) with or without Rg1 (180 µg/mL) treatment; Table S1: Average survival rate of ginsenoside monomers Rb1, Rb2, Rg1, Rg2, Rg3, Rd, Rh1, and Rh2 (all at 180 µg/mL) in extending the CLS of yeast cells; Table S2: List of all proteins displayed in the protein interaction network.
