# Supplementary figures and images for: Ginsenoside Rg1 Delays Chronological Aging in a Yeast Model via CDC19- and SDH2-Mediated Cellular Metabolism
Source: Antioxidants (Basel). 2023 Jan 28;12(2):296. doi: 10.3390/antiox12020296 (PMC9952469; doi:10.3390/antiox12020296)

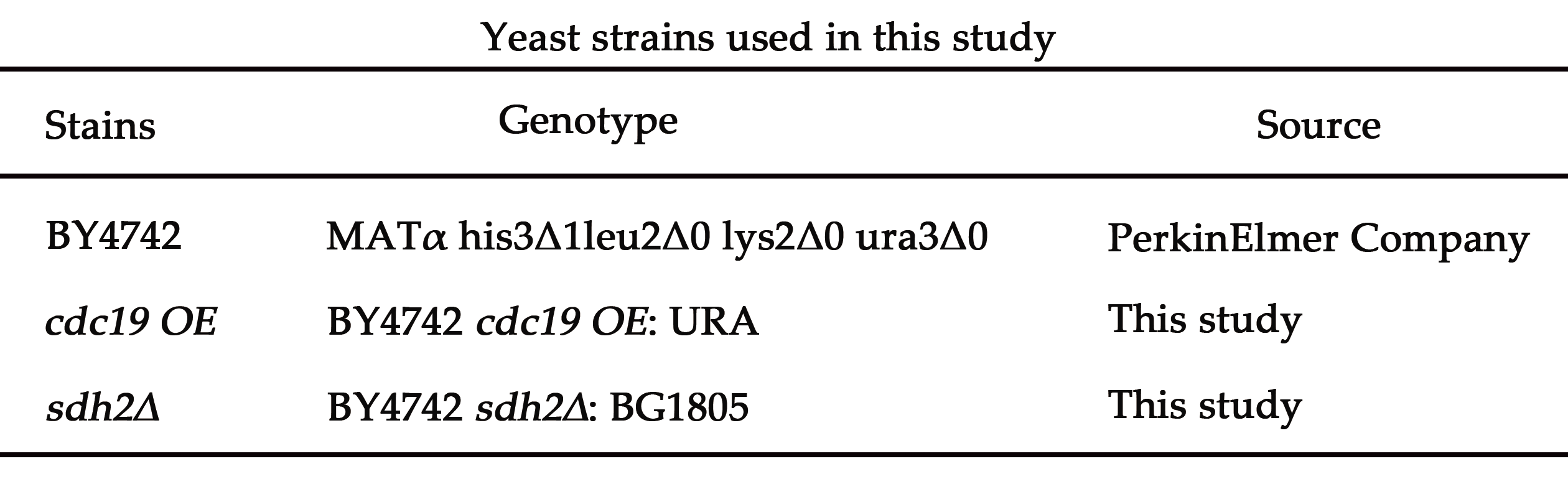

Supplement: Supplementary file 1 [file antioxidants-12-00296-s001.zip › antioxidants-2070108-supplementary/Supplementary materials/Figures/Supplementary Figure 1.tif]

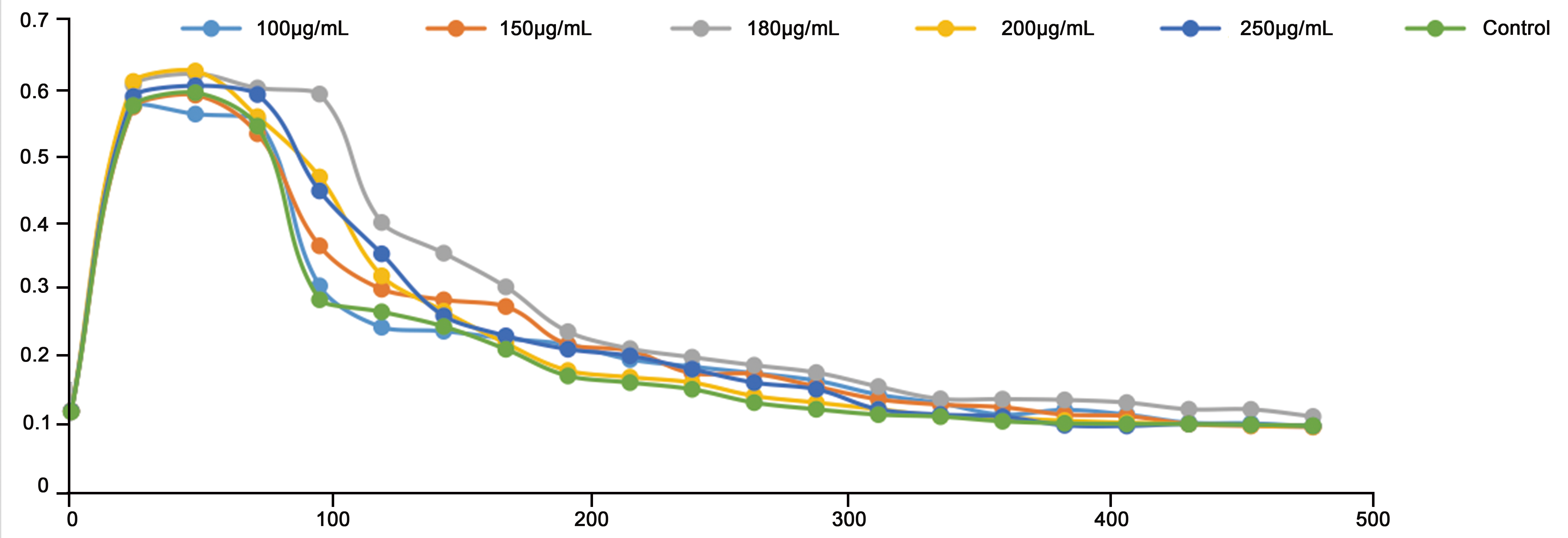

Supplement: Supplementary file 1 [file antioxidants-12-00296-s001.zip › antioxidants-2070108-supplementary/Supplementary materials/Figures/Supplementary Figure 2.tif]

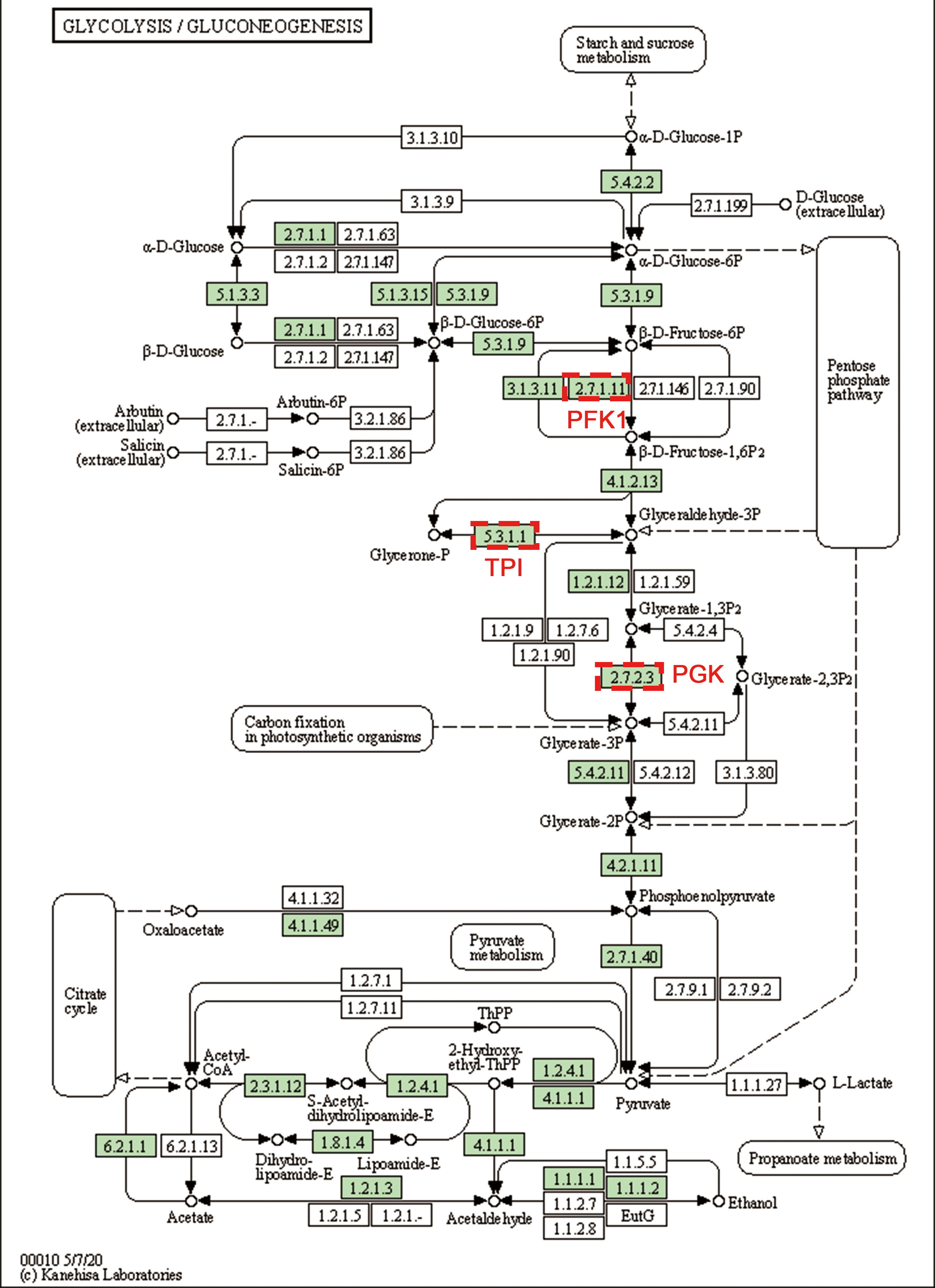

Supplement: Supplementary file 1 [file antioxidants-12-00296-s001.zip › antioxidants-2070108-supplementary/Supplementary materials/Figures/Supplementary Figure 4.tif]

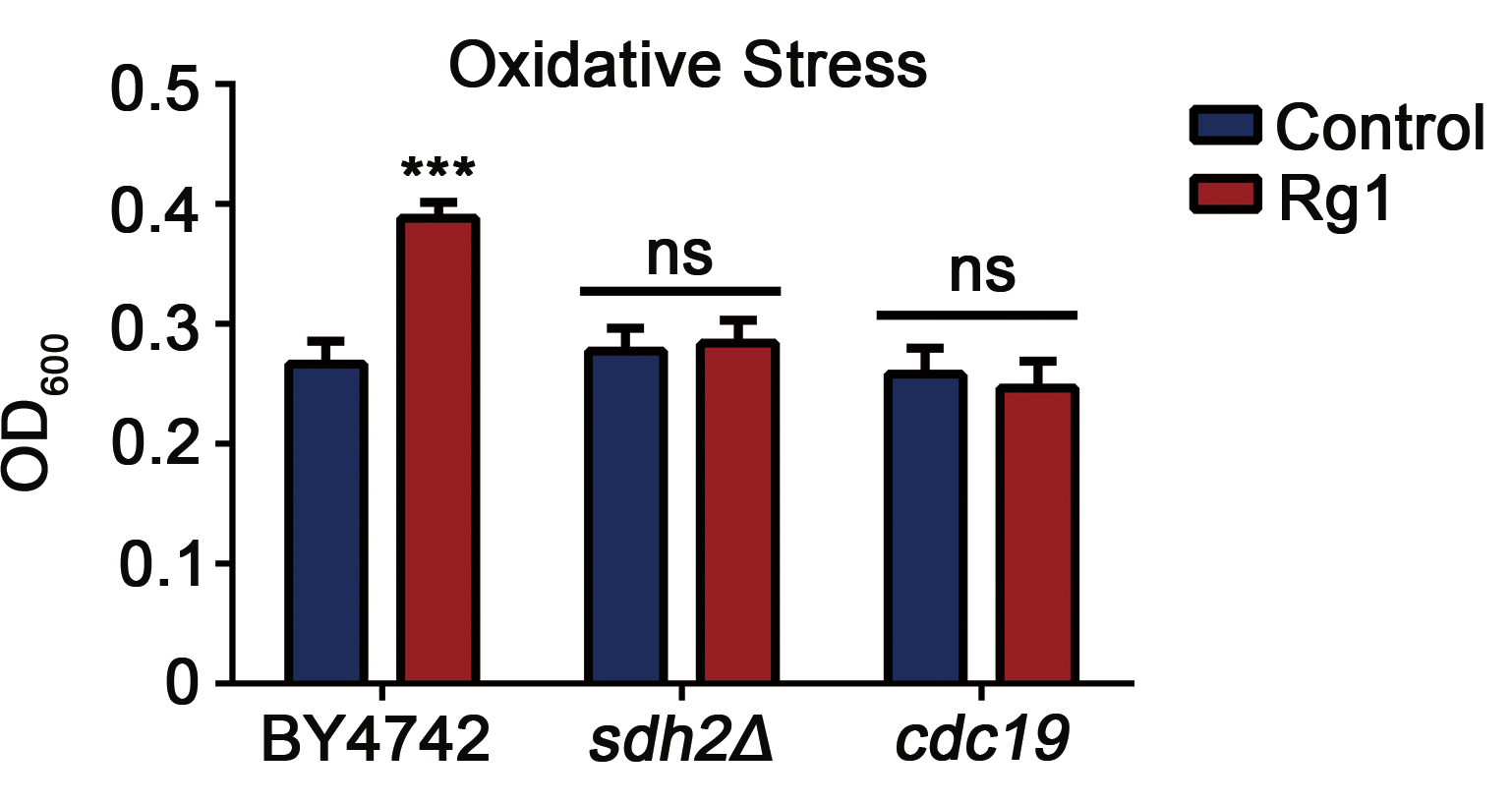

Supplement: Supplementary file 1 [file antioxidants-12-00296-s001.zip › antioxidants-2070108-supplementary/Supplementary materials/Figures/Supplementary Figure 5.tif]

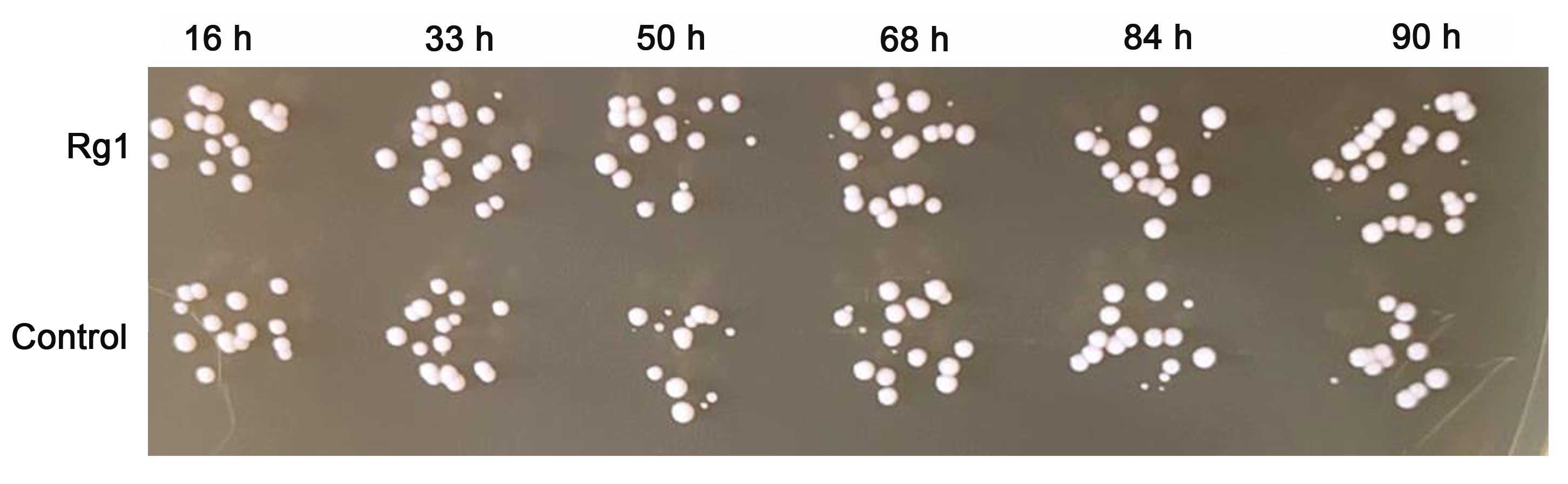

Supplement: Supplementary file 1 [file antioxidants-12-00296-s001.zip › antioxidants-2070108-supplementary/Supplementary materials/Figures/Supplementary Figure 6.tif]

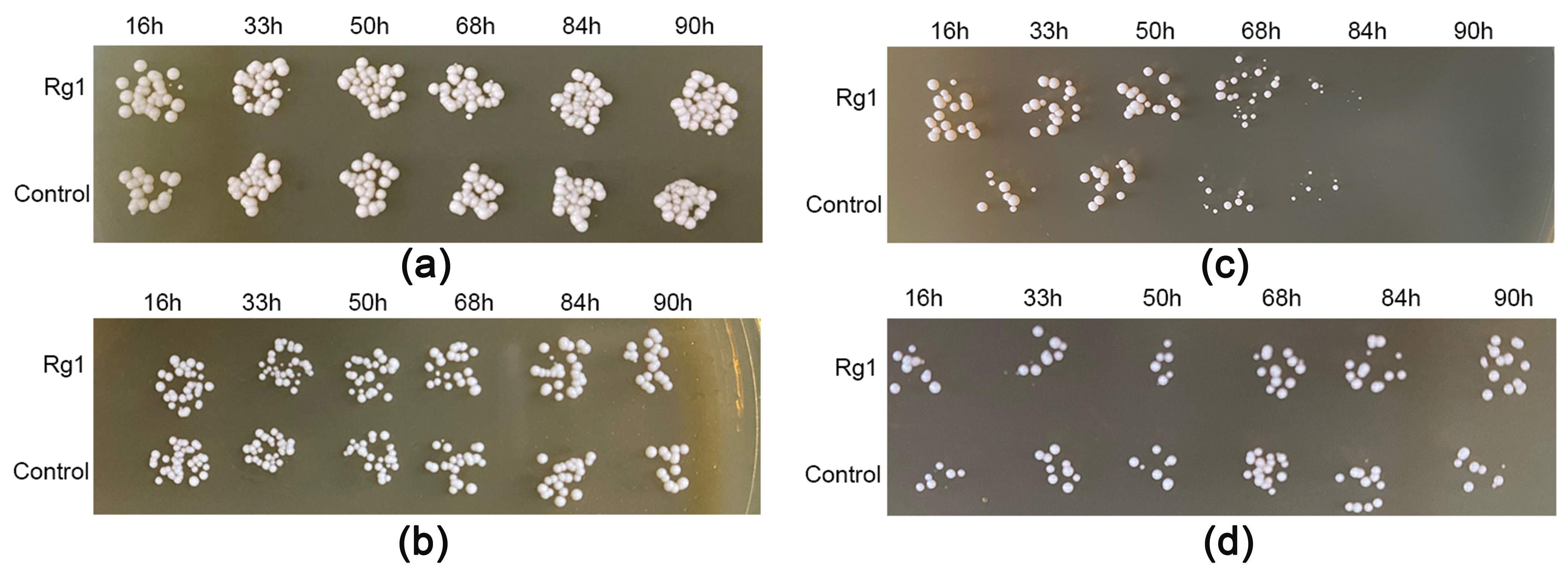

Supplement: Supplementary file 1 [file antioxidants-12-00296-s001.zip › antioxidants-2070108-supplementary/Supplementary materials/Figures/Supplementary Figure 7.tif]
